# Evaluation of tau deposition using ^18^F-PI-2620 PET in MCI and early AD subjects—a MissionAD tau sub-study

**DOI:** 10.1186/s13195-022-01048-x

**Published:** 2022-07-27

**Authors:** Santiago Bullich, Andre Mueller, Susan De Santi, Norman Koglin, Stephen Krause, June Kaplow, Michio Kanekiyo, Núria Roé-Vellvé, Audrey Perrotin, Aleksandar Jovalekic, David Scott, Michelle Gee, Andrew Stephens, Michael Irizarry

**Affiliations:** 1 Life Molecular Imaging GmbH, Berlin, Germany; 2 Eisai Inc, Nutley, NJ USA; 3 Clario, San Mateo, CA USA; 4 Eisai Limited, Hatfield, UK

**Keywords:** PI-2620, Florbetaben, Tau, Amyloid-beta, Mild cognitive impairment, Alzheimer’s disease

## Abstract

**Background:**

The ability of ^18^F-PI-2620 PET to measure the spatial distribution of tau pathology in Alzheimer’s disease (AD) has been demonstrated in previous studies. The objective of this work was to evaluate tau deposition using ^18^F-PI-2620 PET in beta-amyloid positive subjects with a diagnosis of mild cognitive impairment (MCI) or mild AD dementia and characterize it with respect to amyloid deposition, cerebrospinal fluid (CSF) assessment, hippocampal volume, and cognition.

**Methods:**

Subjects with a diagnosis of MCI due to AD or mild AD dementia and a visually amyloid-positive ^18^F-florbetaben PET scan (*n*=74, 76 ± 7 years, 38 females) underwent a baseline ^18^F-PI-2620 PET, T1-weighted magnetic resonance imaging (MRI), CSF assessment (Aβ42/Aβ40 ratio, p-tau, t-tau) (*n*=22) and several cognitive tests. A 1-year follow-up ^18^F-PI-2620 PET scans and cognitive assessments were done in 15 subjects.

**Results:**

Percentage of visually tau-positive scans increased with amyloid-beta deposition measured in ^18^F-florbetaben Centiloids (CL) (7.7% (<36 CL), 80% (>83 CL)). ^18^F-PI-2620 standardized uptake value ratio (SUVR) was correlated with increased ^18^F-florbetaben CL in several regions of interest. Elevated ^18^F-PI-2620 SUVR (fusiform gyrus) was associated to high CSF p-tau and t-tau (*p*=0.0006 and *p*=0.01, respectively). Low hippocampal volume was associated with increased tau load at baseline (*p*=0.006 (mesial temporal); *p*=0.01 (fusiform gyrus)). Significant increases in tau SUVR were observed after 12 months, particularly in the mesial temporal cortex, fusiform gyrus, and inferior temporal cortex (*p*=0.04, *p*=0.047, *p*=0.02, respectively). However, no statistically significant increase in amyloid-beta load was measured over the observation time. The MMSE (Recall score), ADAS-Cog14 (Word recognition score), and CBB (One-card learning score) showed the strongest association with tau deposition at baseline.

**Conclusions:**

The findings support the hypothesis that ^18^F-PI-2620 PET imaging of neuropathologic tau deposits may reflect underlying neurodegeneration in AD with significant correlations with hippocampal volume, CSF biomarkers, and amyloid-beta load. Furthermore, quantifiable increases in ^18^F-PI-2620 SUVR over a 12-month period in regions with early tau deposition are consistent with the hypothesis that cortical tau is associated with cognitive impairment. This study supports the utility of ^18^F-PI-2620 PET to assess tau deposits in an early AD population. Quantifiable tau load and its corresponding increase in early AD cases could be a relevant target engagement marker in clinical trials of anti-amyloid and anti-tau agents.

**Trial registration:**

Data used in this manuscript belong to a tau PET imaging sub-study of the elenbecestat MissionAD Phase 3 program registered in ClinicalTrials.gov (NCT02956486; NCT03036280).

**Supplementary Information:**

The online version contains supplementary material available at 10.1186/s13195-022-01048-x.

## Background

Abnormal accumulation of misfolded extracellular amyloid-beta and intracellular neurofibrillary tangles of tau proteins, associated with synaptic disruption and subsequent neuronal death, characterizes Alzheimer's disease (AD). As AD progresses, amyloid-beta plaques and neurofibrillary tangles follow a distinct pattern of cortical spread across brain regions. Tau pathology presumably accumulates initially in the medial temporal lobe and then spreads throughout the neocortex [1]. As expected from neuropathological data [2], tau positron emission tomography (PET) signal is associated to brain hypometabolism [3], atrophy [4], and cognitive dysfunctions [5] more precisely than amyloid-beta PET. Tau PET signal also closely correlates with total tau (t-tau) and phosphorylated tau (p-tau) concentrations in the cerebrospinal fluid (CSF) [6, 7]. Further, previous studies provide evidence for sequential changes in preclinical AD from amyloidosis to tauopathy to cognitive deficits. Amyloid accumulation precedes tau and seems to accelerate neocortical tau pathology [8].

Several PET probes allowing in vivo tau pathology visualization and quantification have been discovered and are undergoing human evaluation [9–12]. One of these tau tracers (flortaucipir) was approved by the Food and Drug Administration (FDA) for the detection of advanced stages of tau deposition (B3). ^18^F-PI-2620 is a next-generation tau PET tracer with a high binding affinity for pathological tau depositions and low off-target binding for an improved detection of tau deposition at earlier stages [13]. The ability of ^18^F-PI-2620 to measure the spatial distribution of tau pathology in AD has been demonstrated previously [14–16]. However, the characterization of tracer performance in the early stages of tau deposition is critical. With the negative outcomes of several therapeutic clinical trials in reducing the cognitive decline in mild-to-moderate AD [17, 18], investigators were encouraged to explore treatment effects in the earliest possible phase of biomarkers abnormalities event at the asymptomatic stage. PET tracers sensitive to early stages of tau deposition could assist in the detection of at-risk individuals, defining targets for preventive interventions, and aiding clinical trial design focused on providing interventions at the earliest stage possible. Indeed, several studies have used tau as a biomarker for reduction of amyloid-beta [19] and the TRAILBLAZER-ALZ study (NCT04437511), for the first time, used tau PET imaging as an inclusion criterion of a clinical trial of an anti-amyloid monoclonal antibody [20].

In this context, ^18^F-PI-2620 was included into a sub-study of the MissionAD trial of elenbecestat in order to explore the usefulness of tau PET imaging in this specific patient population. The objective of this work was therefore to evaluate tau deposition using ^18^F-PI-2620 PET in amyloid-beta positive subjects with a diagnosis of mild cognitive impairment (MCI) due to AD or mild AD dementia and characterize it with respect to amyloid-beta deposition, CSF (amyloid-beta, t-tau, p-tau), hippocampal volume, and several neurocognitive domains from a series of cognitive instruments.

## Materials and methods

### Study design and participants

The study population consisted of subjects with a clinical diagnosis of MCI due to AD (*n*=72) or mild AD dementia (*n*=2) from the elenbecestat MissionAD Phase 3 program (NCT02956486; NCT03036280) who joined an ^18^F-PI-2620 tau PET imaging sub-study at selected sites in the United States. Inclusion criteria of the sub-study included Mini-Mental State Examination (MMSE) score equal to or higher than 24, Clinical Dementia Rating (CDR) global score of 0.5, CDR Memory Box score equal or higher than 0.5, impaired episodic memory deficit confirmed by a list learning task, and an amyloid-positive ^18^F-florbetaben PET scan by central visual assessment.

Before treatment randomization, all subjects recruited in this MissionAD tau sub-study underwent a baseline ^18^F-PI-2620 PET, T1-weighted magnetic resonance imaging (MRI), and cognitive testing (International Shopping List Test (ISLT), Cogstate Brief Battery (CBB), CDR, MMSE, Alzheimer Disease Assessment Scale-Cognitive14 (ADAS-Cog14), and Functional Activities Questionnaire (FAQ)). CSF assessment (Aβ42/Aβ40 ratio, p-tau, t-tau) was performed in a subset of subjects at baseline (*n*=22). A 1-year follow-up including a ^18^F-PI-2620 PET (*n*=15), ^18^F-florbetaben PET (*n*=15), and cognitive assessments (CDR, MMSE, ADAS-Cog14, and FAQ) (*n*=36) was done in subjects from the placebo arm.

### Image acquisition and reconstruction

Tau PET acquisition consisted of a 30-min PET scan (6 × 5 min dynamic frames) starting at 60 ± 1 min after intravenous injection of 185 MBq ± 20% of ^18^F-PI-2620 followed by a 10 mL saline flush. Amyloid PET acquisition consisted of a 20-min PET scan (4 × 5 min dynamic frames) starting at 90 min after intravenous injection of 300 MBq ± 20% of ^18^F-florbetaben followed by a 10-mL saline flush.

A 3D Hoffmann brain phantom acquired prior to subject enrolment was used to establish a standardized acquisition and reconstruction method to ensure comparability of quantitative PET between imaging sites. PET scans were reconstructed using Ordered Subsets Expectation Maximization (OSEM) algorithm (4 iterations and 16 subsets, zoom = 2) or comparable reconstruction as guided by the Hoffman phantom. PET scans were corrected for attenuation, scatter, randoms, and dead time. A Gaussian smoothing kernel to bring the PET images to a standard spatial resolution (6.5 mm in plane and 7 mm axial) was determined for each scanner using previously acquired Hoffman brain phantoms [21]. Subsequently, a Gaussian smoothing kernel was applied to all the ^18^F-florbetaben and ^18^F-PI-2620 PET images prior to image analysis.

### Image analysis

Image analysis of ^18^F-PI-2620 and ^18^F-florbetaben PET scans was conducted using SPM8 software (https://www.fil.ion.ucl.ac.uk/spm/software/spm8/). Motion correction was performed on each PET frame, and an average PET image was generated. Then, the average PET scan was co-registered to its associated T1-weighted MRI scan. Subsequently, the MRI image was segmented into gray matter, white matter, and cerebrospinal fluid, and spatially normalized to the standard MNI (Montreal Neurological Institute) space. The normalization transformation was applied to the co-registered PET scans and gray matter probability maps. Regions of interest (ROIs) for ^18^F-PI-2620 were defined as the intersection between the standard Automated Anatomic Labeling (AAL) atlas [22] and the normalized gray matter segmentation map thresholded at a probability level of 0.2. ROIs included the frontal, lateral temporal (inferior and superior), occipital, parietal, anterior cingulate, posterior cingulate, fusiform gyrus, and mesial temporal (amygdala, hippocampus, and parahippocampus) cortices and cerebellar gray matter. Mean radioactivity values were obtained from each ROI without correction for partial volume effects applied to the PET data. Regional standardized uptake value ratio (SUVR) was calculated as the ratio of the activity in the target ROI to the activity in the reference region. ^18^F-PI-2620 SUVR images were generated by dividing each voxel activity by the activity in the reference region. For ^18^F-PI-2620 PET scans, cerebellar gray matter excluding the vermis and anterior cerebellar gray matter contiguous to the vermis was used as the reference region [14,15].

Centiloid (CL) values were calculated for each ^18^F-florbetaben PET using the method described by Klunk et al. [23]. ROIs downloaded from the Global Alzheimer’s Association Interactive Network (GAAIN) website (http://www.gaain.org) for the cerebral cortex and the whole cerebellum were applied to the normalized ^18^F-florbetaben PET. Cortical SUVR was calculated as the ratio of the activity in the cortex to the activity in the reference region ROI (whole cerebellum). Finally, the CL values were calculated (CL =153.4 · SUVR − 154.9) [24]. The in-house implementation of the standard CL analysis was previously validated using data freely accessible at the GAAIN website (http://www.gaain.org) [25].

### Visual assessment of ^18^F-PI-2620 PET scans

**Fig. 1:**
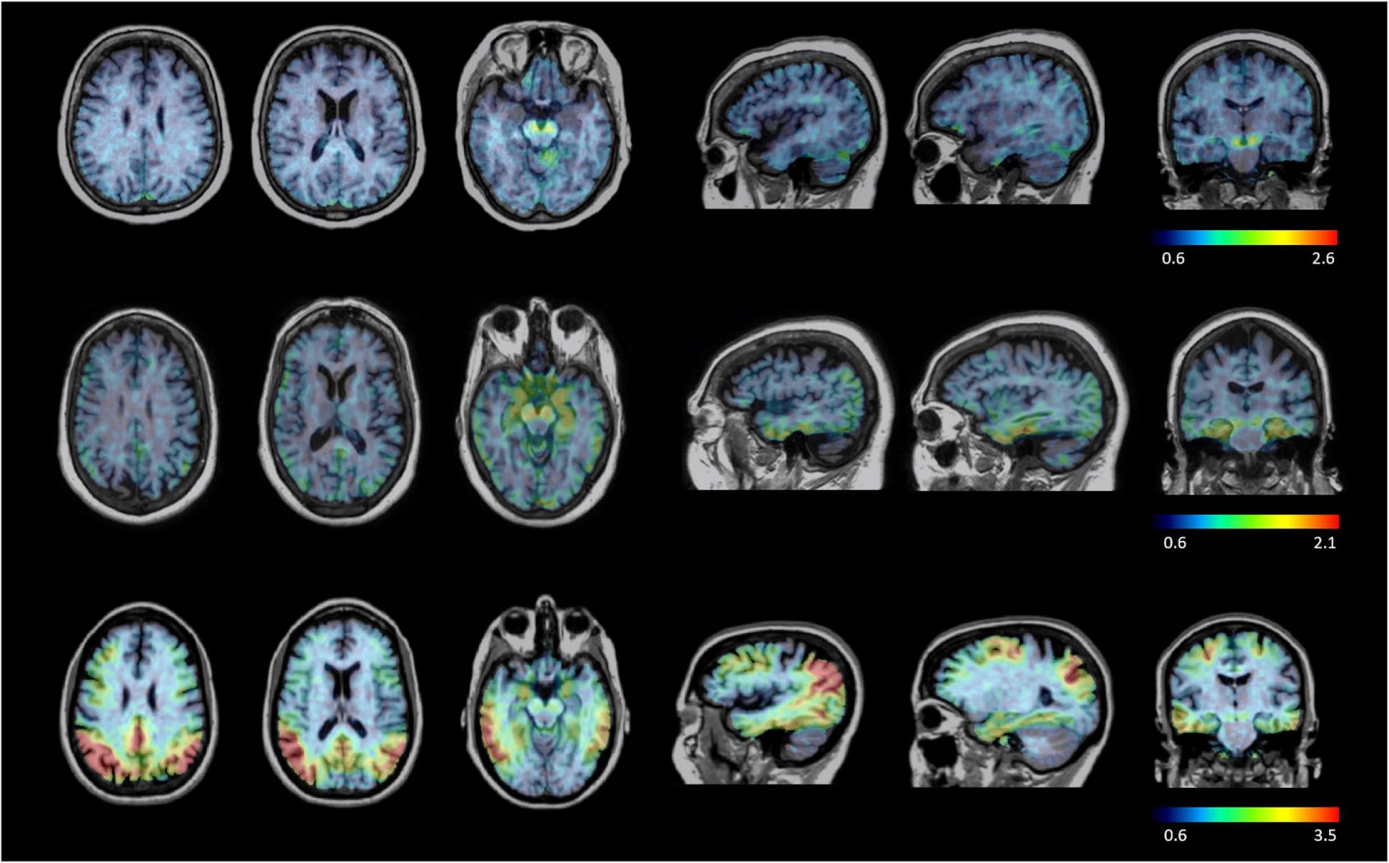
^18^F-PI-2620 SUVR PET images (scalp stripped) registered to the T1-weighted MRI illustrating the tracer distribution in a tau-negative subject (top row), a tau-positive subject with uptake in the mesial temporal cortex (center row), and a tau-positive subject with extensive neocortical uptake (bottom row)

Visual assessment of the ^18^F-PI-2620 PET images was based on the consensus of three readers. Visual assessment was performed on ^18^F-PI-2620 SUVR images registered to the subject’s T1-weighted MRI scans. The readers were allowed to assess any orientation of the ^18^F-PI-2620 SUVR PET image and change its transparency to identify the anatomical location of the tracer retention on the registered T1-weighted MRI. Visual assessment was performed to identify cortical areas of tracer retention above the activity in the reference region (inferior cerebellum). The ^18^F-PI-2620 PET scans were considered tau positive if at least one cortical region, either unilateral or bilateral, was positive. In case of low assessment confidence, the readers used the SUVR value(s) of the region(s) as an adjunct to the visual assessment. SUVR values larger than two standard deviations above the mean SUVR of the healthy controls, as reported in Mueller et al., were considered as signs of positivity [14]. Figure 1 shows an illustrative example of the tracer distribution in a tau-negative healthy control, a tau-positive subject with predominant specific uptake in the mesial temporal cortex, and a tau-positive subject with extensive neocortical tracer retention.

### Hippocampal volume

Hippocampal volume was derived from 3D T1-weighted images acquired at 1.5T and 3T, consisting of sagittal 3D magnetization-prepared rapid gradient-echo (Siemens), sagittal 3D turbo field echo (Philips), coronal 3D fast spoiled gradient recalled (General Electric), or sagittal 3D field echo (Toshiba) sequences with 1.2 mm-thick slices and a 1.25 × 1.25-mm^2^ in-plane resolution. The images were parcellated using FreeSurfer [26, 27] and the Desikan [28] atlas for hippocampus region-of-interest definition.

### Statistical methods

#### Descriptive statistics

Subject characteristics were summarized using mean, standard deviation, minimum, and maximum for continuous variables, counts, and frequencies for categorical variables. Continuous variables were compared using the Wilcoxon rank sum test and categorical variables were compared using chi-squared test.

#### Association of quantitative amyloid-beta load and visual assessment of the tau load

The percent of tau-positive scans by visual assessment was calculated for low amyloid-beta load subjects (CL<36) based on previously published CL cutoffs [25] and elevated amyloid-beta load subjects (CL>36) split in two parts with an equal number of subjects (CL 36–83 CL, CL>83). Percent of tau-positive cases for the different levels of amyloid-beta were compared statistically using the chi-squared test for trend in proportions.

#### Association between quantitative amyloid-beta and tau load

The association between ^18^F-florbetaben CL and ^18^F-PI-2620 SUVR was assessed by means of scatter plots and the Spearman correlation coefficients (r). Additionally, the subjects were classified according to the amyloid-beta load in three ^18^F-florbetaben CL categories as described above (<36 CL, 36–83 CL, >83 CL). The mean tau load in subjects in the three ^18^F-florbetaben CL categories was compared using analysis of variance (ANOVA). Comparisons across categories were performed using Tukey’s honestly significant difference test. The proportion of tau-positive subjects by quantitative assessment in the three ^18^F-florbetaben CL categories was compared by means of the chi-squared test for trend in proportions. ^18^F-PI-2620 scans were considered tau-positive by quantitative assessment when regional SUVR was two standard deviations above the mean SUVR of the healthy controls reported in Mueller et al. [14].

#### Association between CSF and PET measurements

A linear regression model was fitted to assess the association between CSF Aβ42/Aβ40 ratio versus ^18^F-florbetaben CL and between CSF p-Tau and t-tau versus regional ^18^F-PI-2620 PET.

#### Assessment of the amyloid-beta and tau load over time

To evaluate the amyloid-beta and tau change over time, linear mixed effects models were fitted and spaghetti plots showing individual trajectories per participant generated. The individual percent amyloid-beta and tau deposition over time was assessed as the percent change in SUVR calculated as 100×(SUVR_FU_ – SUVR_B_)/SUVR_B_ where SUVR_B_ and SUVR_FU_ are the SUVR at baseline and follow-up scans, respectively.

#### Assessment of tau load and cognition at baseline

The association between ^18^F-PI-2620 SUVR and cognitive scores at baseline was assessed by means of Spearman’s correlation coefficient and a multivariate linear regression model with a stepwise selection technique. The predictors included in this model for assessment were baseline PI-2620 SUVR, baseline CL, sex, and age. Correlation analysis was performed at the ROI level and voxel-wise level on the images normalized in the MNI space to generate correlation maps. Given the exploratory nature of the analysis, no correction for multiple comparisons was performed.

#### Assessment of tau load at baseline and cognitive decline over time

Linear mixed effects models were fitted to evaluate the cognitive decline over time while correcting for ^18^F-PI-2620 SUVR at baseline.

## Results

### Demographics

Subject characteristics of the population enrolled in the tau PET Imaging sub-study are summarized in Table 1 for those subjects that underwent baseline assessment prior to treatment randomization (*n*=74), and subjects in the placebo arm that underwent longitudinal cognitive assessment (*n*=36) and ^18^F-PI-2620 PET scans (*n*=15). No significant differences were observed across the three groups in terms of age, MMSE (baseline) and CDR-SB (baseline), and CL. The subjects that underwent longitudinal ^18^F-PI-2620 PET scans showed a significantly lower tau-load at baseline in comparison with the remaining subjects.

**Table 1:** Demographic characteristics of the study participants

	Baseline dataset	Subset with longitudinal cognitive information	*p*^1^	Subset with longitudinal PET scans	*p*^2^
*n*	74	36		15	
Age (years)	75.78 ± 6.61 (54, 85)	75.69±7.13 [55, 85]	N.S	75.33 ± 8.92 (57, 85)	N.S
Sex	36 (48.6%) M	17 (47.2%) (M)	N.S	8 (53.3%) M	N.S
	38 (51.4%) F	19 (52.8%) (F)		7 (46.7%) F	
Clinical diagnosis	72 (MCI due to AD)	34 (MCI due to AD)	N.S	14 (MCI due to AD)	N.S
	2 (mild AD)	2 (mild AD)		1 (Mild AD)	
MMSE	26.51 ± 2.5 (20, 30)	26.31±2.56 [21, 30]	N.S	26.93 ± 2.41 (21, 30)	N.S
CDR-SB	2.35 ± 1.04 (0.5, 5)	2.51±1.1 [0.5, 5]	N.S	2.47 ± 0.79 (1, 4)	N.S
CL	71.64±40.07 (−13.39, 181.87)	71.81±36.14 [7.2, 154.75]	N.S	62.16±41.46 (7.2, 154.75)	N.S
SUVR (PI-2620)	1.28±0.35 [0.89, 2.43]	1.31±0.39 [0.89, 2.43]	N.S	1.1±0.17 [0.89, 1.65]	*p*=0.01

*MCI* mild cognitive impairment, *AD* Alzheimer’s disease, *MMSE* Mini-Mental State Examination, *CDR-SB* Clinical dementia rating – Sum of boxes, CL (^18^F-florbetaben) centiloid at baseline, *SUVR(*^*18*^*F-PI-2620)* Standardized uptake value for ^18^F-PI-2620 scans at baseline in the inferior temporal cortex, *p*^1^*p*-values comparing the demographic characteristics between the subset of subjects with longitudinal cognitive information and the remaining subjects from the baseline dataset (Wilcoxon rank sum (continuous variables) test and chi-squared test (categorical variables)), *p*^2^*p*-values comparing the demographic characteristics between the subset of subjects with longitudinal ^18^F-PI-2620 scans and the remaining subjects from the baseline dataset (Wilcoxon rank sum test (continuous variables) and chi-squared test (categorical variables)), N.S non-significant differences (*p*>0.05)

### Association of amyloid-beta load and visual assessment of the tau load

**Fig. 2:**
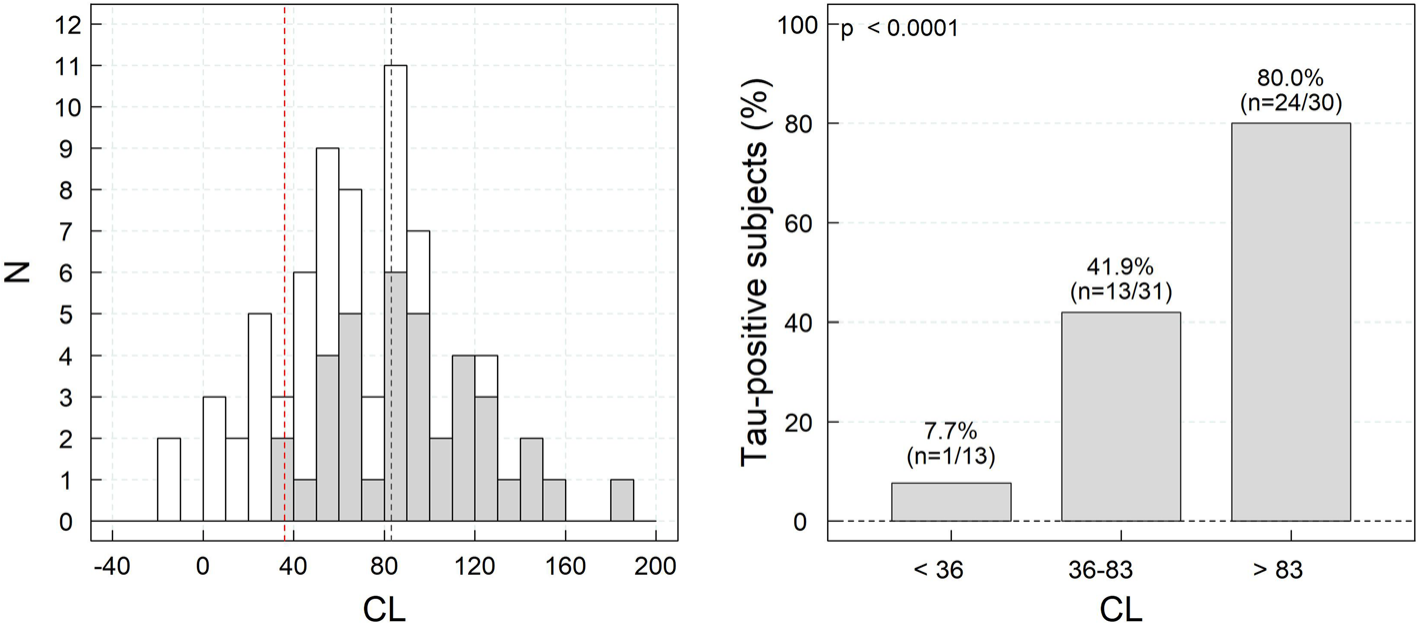
Histogram of the number of subjects across ^18^F-florbetaben CL values (tau-positive subjects are marked in gray; cutoff for established amyloid pathology (36 CL) and high amyloid-load (82 CL) are marked with red and gray dashed lines, respectively) (left panel) and percent of visually tau-positive subjects by amyloid-beta load category (right panel)

In the studied population of amyloid-beta positive subjects by visual assessment, CL values ranged between -13.4 and 181.8. Fifteen subjects had low CL values below 36. These subjects showed regional uptake that visual readers could detect, but this regional PET signal was diluted in the large cortical regions of interest used by the CL method. At baseline, 51% (*n*=38) of the cases were tau-positive by visual assessment. The proportion of visually tau-positive scans increased with the amyloid-beta load (7.7% (<36 CL), 41.9% (36–83 CL), and 80.0% (>83 CL) (*χ*^2^= 20.9, *p*<0.0001) (Fig. 2).

### Association between amyloid-beta load and tau load assessed quantitatively

**Fig. 3:**
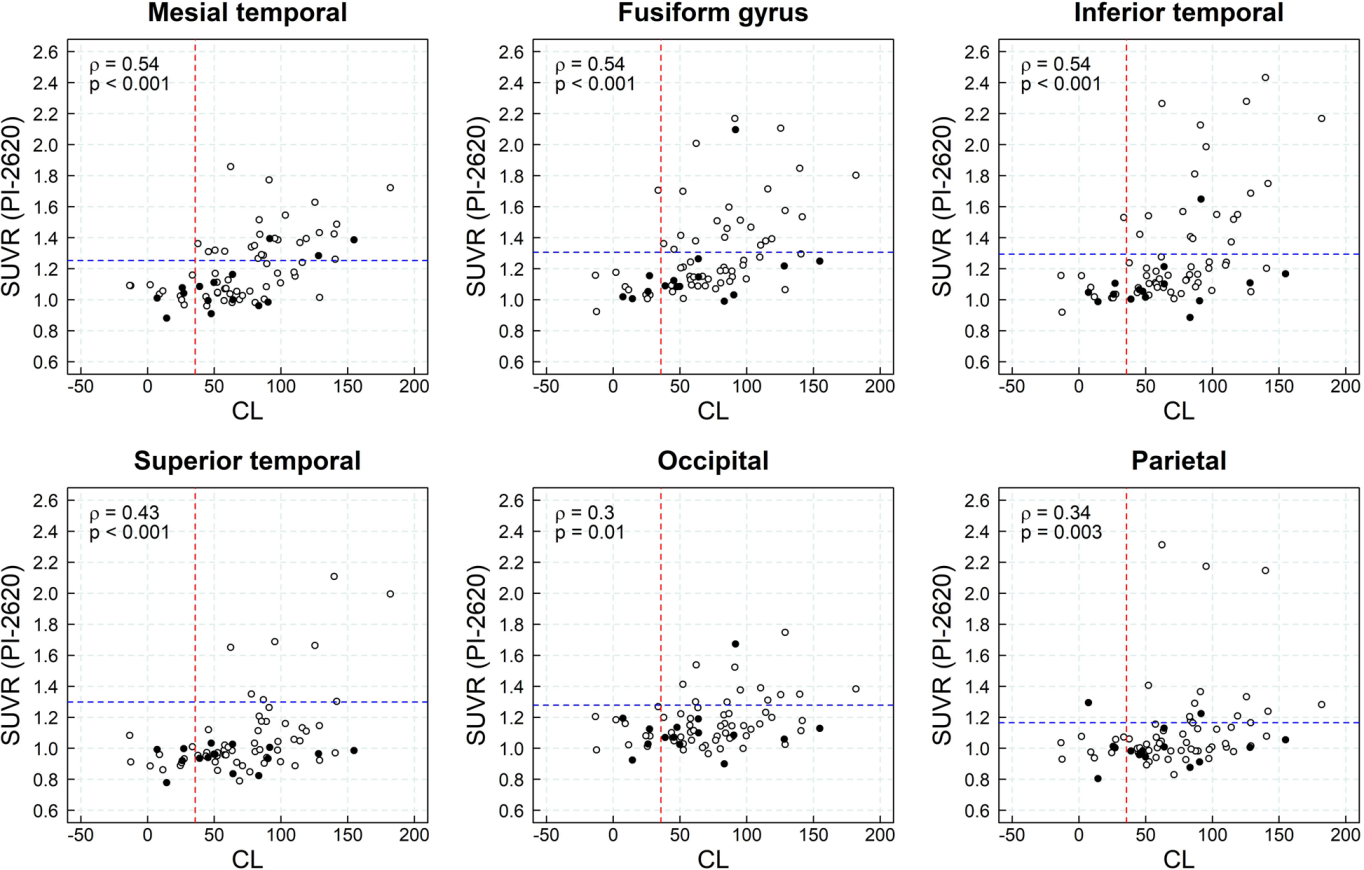
Scatter plots of the ^18^F-PI-2620 SUVR versus amyloid-beta ^18^F-florbetaben CLs. Red and blue lines represent ^18^F-PI-2620 SUVR and ^18^F-florbetaben CL positivity cutoffs, respectively, derived from previous publications [14, 25]. Solid dots correspond to those subjects that underwent 1-year follow-up ^18^F-PI-2620 PET scans

Spearman correlation coefficients between CL and ^18^F-PI-2620 SUVRs ranged from 0.3 (occipital cortex) to 0.54 (mesial temporal cortex, fusiform gyrus, and inferior temporal cortex). Tau deposition was low in subjects with minor amyloid-beta deposits (CL<36), increasing significantly in the mesial temporal cortex, fusiform gyrus, inferior temporal, superior temporal and occipital cortices of subjects with high amyloid-beta load (CL>83) (Fig. 3, Table 2). The percent of tau-positive subjects by quantitative assessment (i.e., ^18^F-PI-2620 SUVR at least 2 SD above the mean SUVR of healthy controls) was significantly higher in subjects with elevated amyloid-beta deposition (CL>36) than in low amyloid-beta deposition (CL<36) (mesial temporal cortex (63.3% (>83CL) vs. 25.8% (36–83CL) vs. 0.0% (<36CL)), fusiform gyrus (53.3% (>83CL) vs. 22.6% (36–83CL) vs. 7.7% (<36CL)), inferior temporal cortex (50% (>83CL) vs. 12.9% (36–83 CL) vs. 0.0% (<36CL)) and occipital cortex (33.3% (>83CL) vs. 9.7% (36–83CL) vs. 0.0% (<36CL))) (Table 3). High agreement was found (85.1%) between visual assessment and global binary quantitative assessment (i.e., at least one region shows ^18^F-PI-2620 SUVR at least 2 SD above the mean SUVR of healthy controls) of ^18^F-PI-2620 PET scans.

**Table 2:** Regional ^18^F-PI-2620 SUVR (mean±SD [minimum, maximum]) by amyloid-beta group

Centiloid	< 36	36-83	*p*^1^	>83	*p*^2^	*p*^3^	*p*^4^
Mesial temporal	1.04±0.07 [0.88, 1.16]	1.13±0.19 [0.91, 1.86]	0.28	1.32±0.21 [0.96, 1.77]	**0.0006**	**0.00006**	**< 0.0001**
Fusiform gyrus	1.11±0.19 [0.92, 1.71]	1.22±0.21 [1.01, 2.01]	0.39	1.43±0.32 [0.99, 2.17]	**0.008**	**0.001**	**0.0005**
Inferior temporal	1.08±0.15 [0.92, 1.53]	1.19±0.24 [1.00, 2.27]	0.60	1.45±0.41 [0.89, 2.43]	**0.004**	**0.002**	**0.0005**
Superior temporal	0.93±0.08 [0.78, 1.08]	1.00±0.16 [0.79, 1.65]	0.68	1.16±0.32 [0.82, 2.11]	**0.02**	**0.01**	**0.004**
Occipital	1.10±0.10 [0.92, 1.27]	1.13±0.13 [0.97, 1.54]	0.84	1.22±0.19 [0.9, 1.75]	**0.05**	**0.05**	**0.02**
Parietal	1.01±0.11 [0.8, 1.29]	1.06±0.26 [0.83, 2.31]	0.84	1.17±0.3 [0.88, 2.17]	0.26	0.18	0.13

*CL*^18^F-florbetaben centiloid, *SUVR* standardized uptake value ratio, *SD* standard deviation, *p*^1^, *p*^2^, and *p*^3^*p*-values obtained from Tukey’s honestly significant difference test comparing <36 vs 36–83, 36–83 vs >82, and <36 vs >83, respectively, *p*^4^*p*-values obtained using the analysis of variance (ANOVA) to compare the mean ^18^F-PI-2620 SUVR in different amyloid-beta levels

**Table 3:** Regional percent of ^18^F-PI-2620-positive subjects by quantitative assessment (i.e., ^18^F-PI-2620 SUVR at least two standard deviations above the mean SUVR in healthy controls) in each amyloid-beta group

^18^F-florbetaben centiloid	< 36	36-83	> 83	*p*
Mesial temporal	0.0% (0/13)	25.8% (8/31)	63.3% (19/30)	**< 0.0001**
Fusiform gyrus	7.7% (1/13)	22.6% (7/31)	53.3% (16/30)	**0.001**
Inferior temporal	7.7% (1/13)	12.9% (4/31)	50% (15/30)	**0.0007**
Superior temporal	0.0% (0/13)	6.5% (2/31)	20% (6/30)	**0.03**
Occipital	0.0% (0/13)	9.7% (3/31)	33.3% (10/30)	**0.003**
Parietal	7.7% (1/13)	9.7% (3/31)	33.3% (10/30)	**0.02**

*p p*-values obtained using the chi-squared test for trend in proportions

### Association between CSF and PET measurements

**Fig. 4:**
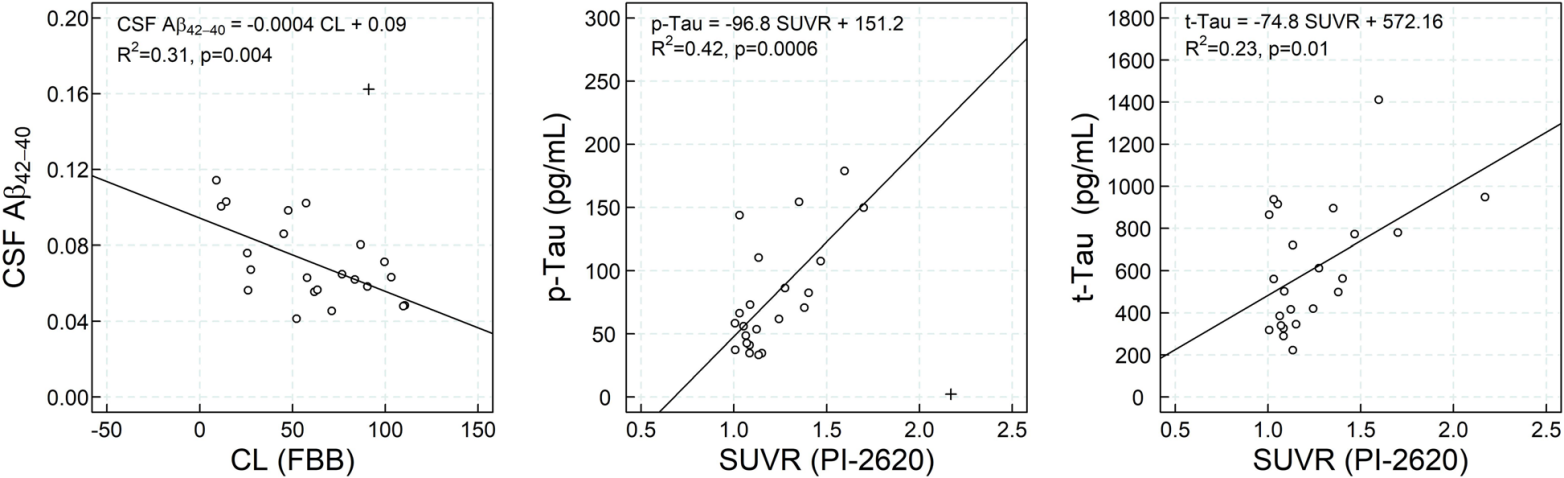
Scatter plot of CSF Aβ42/Aβ40 ratio versus ^18^F-florbetaben CL (left), CSF p-Tau versus ^18^F-PI-2620 SUVR (fusiform gyrus) (center) and CSF t-Tau versus ^18^F-PI-2620 SUVR (fusiform gyrus) (right). The subject marked with a cross was considered an outlier and not included in the linear regression

Elevated ^18^F-florbetaben CL values were associated with lower CSF Aβ42/Aβ40 ratio (CSF_Aβ42/Aβ40_ = −0.0004 CL + 0.09; *R*^2^=0.31, *p*=0.004). Elevated ^18^F-PI-2620 SUVR in the fusiform gyrus was associated with higher levels of CSF p-tau (CSF_p-tau_= −96.8 SUVR + 151.2, *R*^2^=0.42, *p*=0.0006) and t-tau (CSF_t-tau_ = −74.8 SUVR + 572.16, *R*^2^=0.23, *p*=0.01) (Fig. 4).

### Hippocampal volume

Elevated ^18^F-PI-2620 SUVR in the mesial temporal and fusiform gyrus was associated with a reduction in the hippocampal volume (p=0.007, ρ=-0.37 (mesial temporal); p=0.02, ρ=-0.37 (fusiform gyrus) and p=0.12, ρ=-0.37 (inferior temporal)).

### Assessment of the amyloid-beta and tau deposition over time

**Fig. 5:**
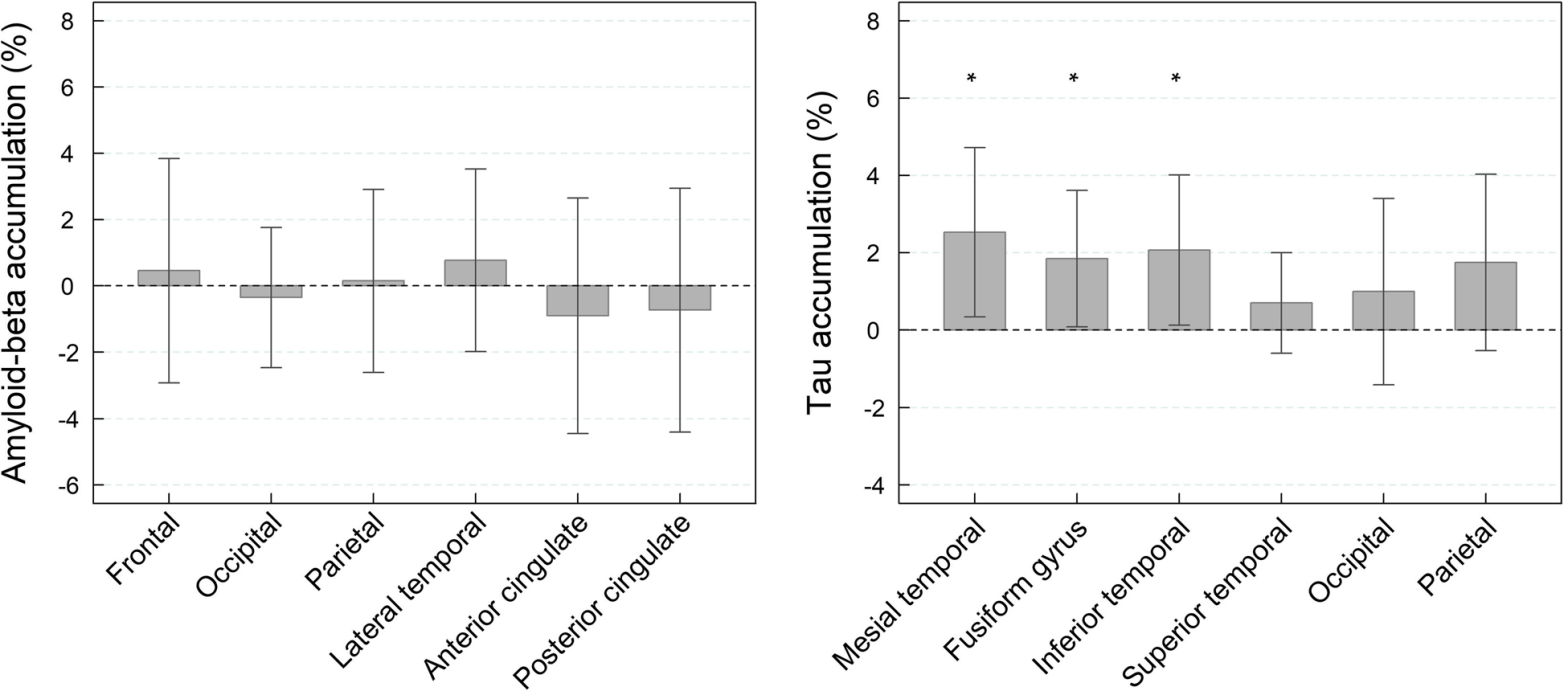
Percent amyloid-beta and tau accumulation (percent SUVR change) over 1-year follow-up. Statistically significant changes (*p*-value < 0.05) obtained from the linear mixed effect model are marked with an asterisk (*)

In the longitudinal subset, 15 subjects underwent ^18^F-florbetaben, and ^18^F-PI-2620 PET scans both at baseline and 1-year follow-up (371±51 days (18F-florbetaben), 353±47 days (^18^F-PI-2620)). Statistically significant increase in tau SUVR was detected in the mesial temporal cortex (2.53 ± 4.32%, *p*=0.04), fusiform gyrus (1.85 ± 3.49%, *p*=0.048), and inferior temporal cortex (2.07 ± 3.84%, *p*=0.04) (Fig. 5, Table 4). Supplemental material 1 shows the individual trajectories per participant stratified according to their amyloid level. Noticeably, most of the cases with longitudinal scans had low tau uptake at baseline, indicating the ability of ^18^F-PI-2620 PET scans to detect tau increase in this early population. No statistically significant increase in amyloid-beta SUVR was observed in any of the cortical regions analyzed (*p* > 0.05) (Fig. 5, Table 4).

**Table 4:** Percent amyloid-beta and tau accumulation over 1 year and *p*-values assessing significant accumulation over time

	Region	Percent SUVR change	*p*
Amyloid-beta	Frontal	0.46 ± 6.68 %	0.70
	Occipital	− 0.35 ± 4.17 %	0.90
	Parietal	0.16 ± 5.44 %	0.72
	Lateral temporal	0.77 ±5.44 %	0.63
	Anterior cingulate	− 0.90 ± 7.01 %	0.88
	Posterior cingulate	− 0.73 ± 7.26 %	0.86
Tau	Mesial temporal	2.53 ± 4.32 %	**0.04**
	Fusiform gyrus	1.85 ± 3.49 %	**0.05**
	Inferior temporal	2.07 ± 3.84 %	**0.04**
	Superior temporal	0.70 ± 2.57 %	0.31
	Occipital	1.00 ± 4.76 %	0.28
	Parietal	1.75 ± 4.51 %	0.10

*SUVR* standardize uptake value ratio, *p*: *p*-values obtained using linear mixed effect models

### Assessment of tau load and cognition at baseline

**Fig. 6:**
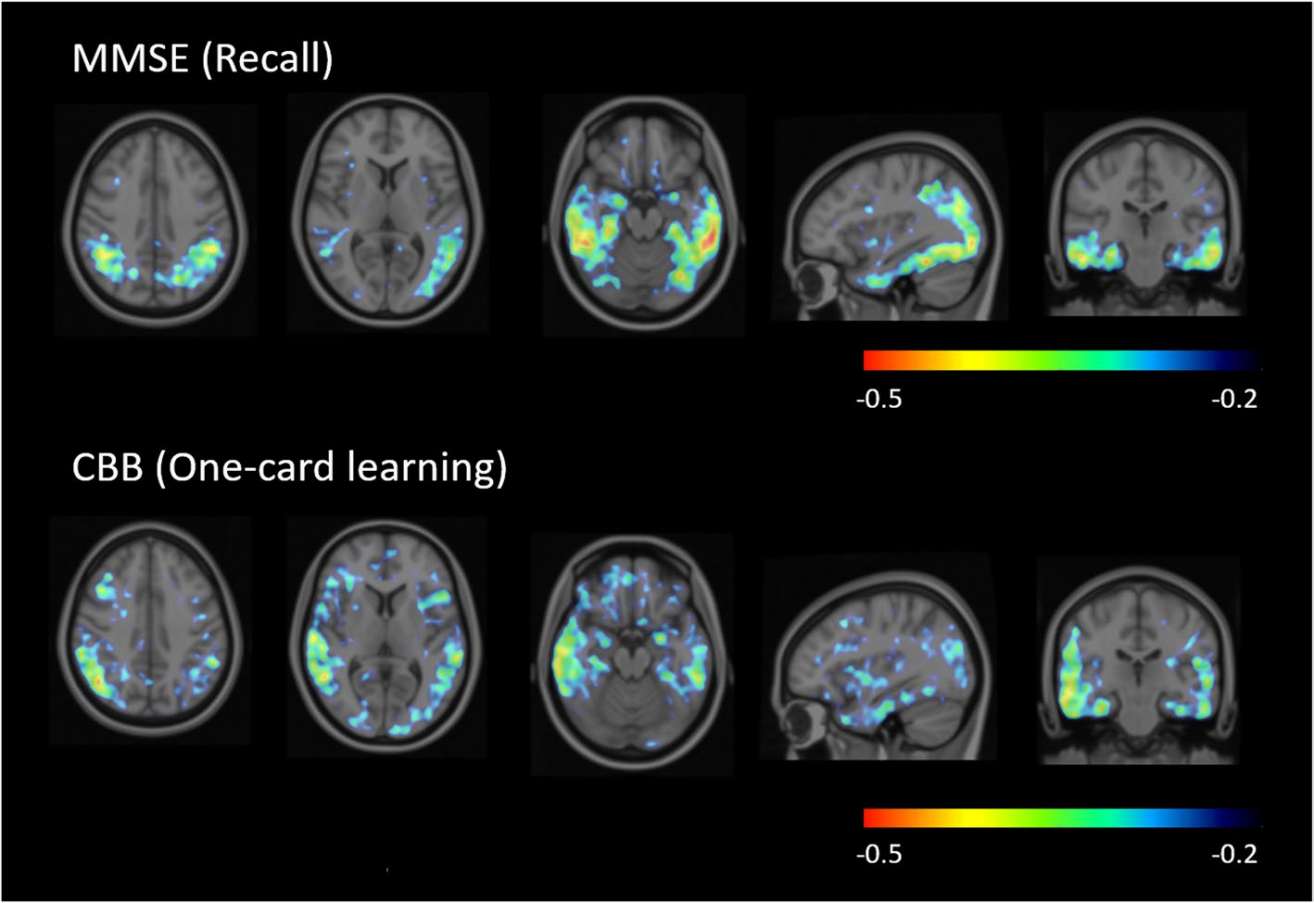
Voxel-wise spearman correlation maps assessing the association between ^18^F-PI-2620 deposition and cognition at baseline displayed on top of the SPM’s T1-weighted template in the MNI space

**Fig. 7:**
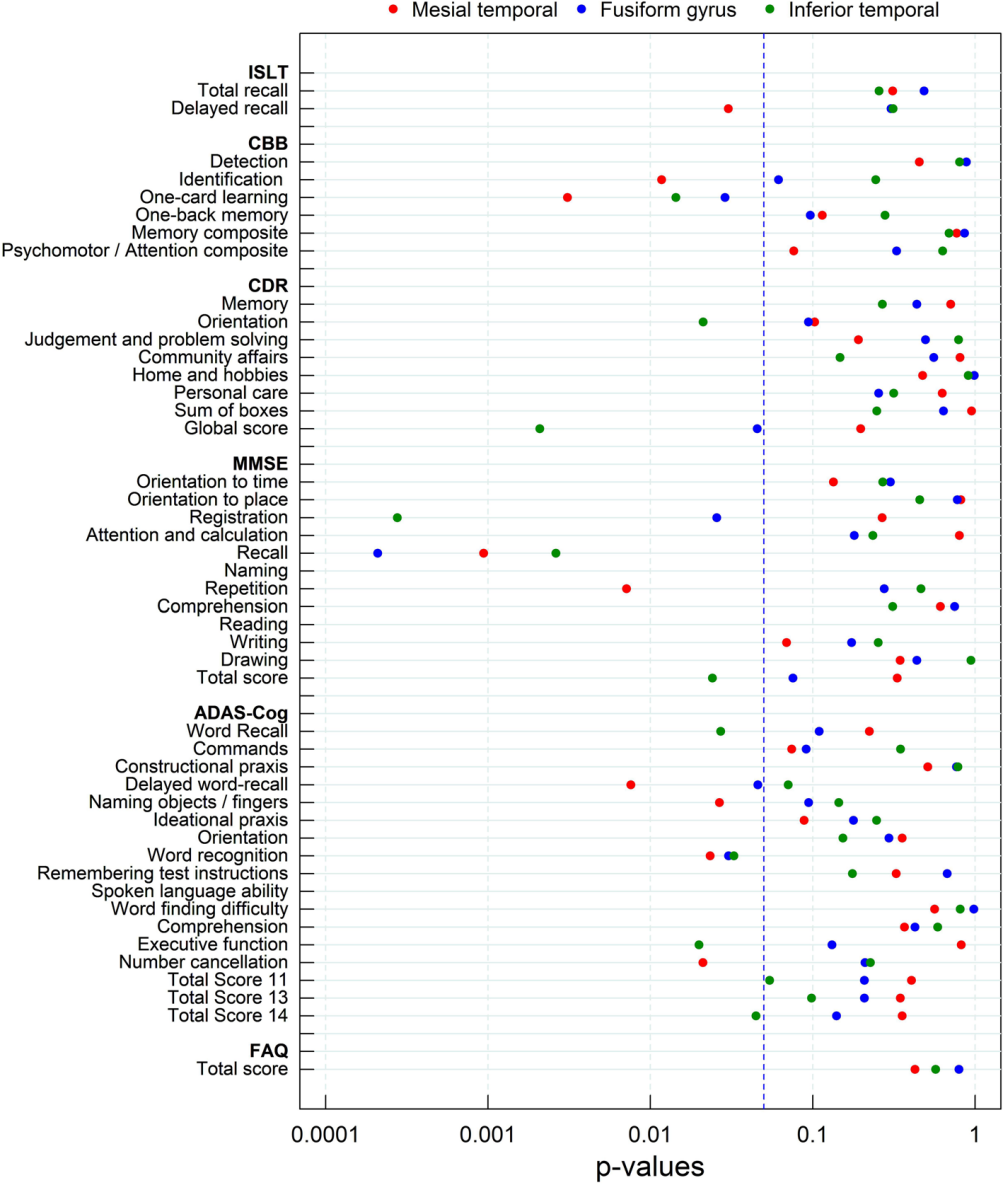
*P*-values assessing the association of ^18^F-PI-2620 deposition and cognition at baseline

In this population, association between tau SUVR and cognitive scores at baseline was mainly found in the mesial temporal cortex, fusiform gyrus, and inferior temporal cortex. The MMSE recall score and CBB one-card learning score showed the strongest association with tau deposition at baseline with Spearman correlation coefficients of − 0.37 and −0.33 (mesial temporal), −0.40 and −0.30 (fusiform gyrus), and −0.42 and −0.33 (inferior temporal cortex), respectively, Similar results were obtained using voxel-wise correlation maps with regional Spearman correlation coefficients (Fig. 6). The MMSE recall score, ADAS-Cog word recognition score, and CBB one-card learning score showed consistent *p*-values below 0.05 (without correction for multiple comparisons) in these three regions (Fig. 7, supplemental material 2). Other cognitive assessments that showed *p*-values below 0.05 (without correction for multiple comparisons) in some regions were ISLT delayed recall, CBB identification, CDR orientation and global scores, MMSE registration, repetition and total scores, ADAS-Cog word recall, delayed word recall, naming objects/fingers executive function, number cancellation, and total scores (Fig. 7).

### Assessment of tau load at baseline and cognitive decline over time

No significant association was found between tau load at baseline and cognitive decline over time.

## Discussion

^18^F-PI-2620 is a PET tracer developed to assess tau deposition in the brain with high sensitivity and low off-target binding. Excellent in-vitro characteristics, dosimetry, test-retest, and in vivo tracer kinetics have been shown [13, 15]. Initial clinical data demonstrated a high image quality for imaging tau deposition in AD subjects [14, 16]. The current work has characterized the tau deposition using ^18^F-PI-2620 PET tracer in the early stages of the AD continuum which corresponds to a therapeutically relevant population. In this population, tau deposition was mainly observed in subjects with established amyloid pathology. Tau deposition was infrequent in subjects with lower amyloid-beta load. These results are in agreement with previous evidence that cortical amyloid-beta is correlated with the spread of tau outside the mesial temporal region [29]. In this study, only 1 out of 13 subjects (7.7%) with less than 36 CL was visually tau positive while 21 out 30 subjects (80%) with greater than 83 CL of amyloid showed ^18^F-PI-2620 accumulation based on visual assessment. Additionally, similar bivariate associations between amyloid-beta and tau have been reported for other PET tracers: ^18^F-florbetaben and ^18^F-flortaucipir [25], ^18^F-NAV4694 and ^18^F-MK6240 [30], 11C-PiB and ^18^F-flortaucipir [31], and ^18^F-florbetapir and ^18^F-flortaucipir [5]. Tau deposition was predominantly present in the mesial temporal, fusiform gyrus, and inferior temporal cortex and less frequent in the occipital and parietal cortices, as expected in the population included in this study. This finding was in concordance with the expected spatiotemporal patterns of tau deposition in the brain reflected in the Braak and Delacourte staging [32, 33]. In addition, high ^18^F-PI-2620 retention was associated with higher CSF p-tau and t-tau and lower hippocampal volume, as previously reported for other tau tracers [4, 6, 7, 34]. ^18^F-PI-2620 is not yet validated using histopathological confirmation of tau load in the brain. However, current results show that ^18^F-PI-2620 can detect tau deposition in the early population included in the study, and tracer deposition as measured by tau PET imaging is concordant with the expected pattern of neurofibrillary tangle accumulation in the brain. Furthermore, the ^18^F-PI-2620 accumulation correlates with other biomarkers associated with tau buildup in AD.

In the longitudinal evaluation, a statistically significant increase in tau deposition over 1 year was observed in the regions with the highest tau burden at baseline (mesial temporal, fusiform gyrus, and inferior temporal cortex). As an exploratory analysis, the assessment of tau deposition over time was expanded by including longitudinal ^18^F-PI-2620 PET of those subjects treated with elenbecestat (*n*=12). Due to the early termination of the Mission-AD program because of an unfavorable risk-benefit ratio including no evidence of potential efficacy, it was hypothesized that elenbecestat may not to have any effect on tau deposition and cognition. The results confirmed tau deposition over 1 year followed a similar pattern as observed in subjects under placebo (Supplemental material 3). These results suggest a different rate of tau accumulation as a function of SUVR at baseline, such that the regions affected earlier in the MCI population are those that accumulate more tau. This pattern of changes is consistent with other longitudinal tau PET studies showing different accumulation rates depending on the tau load at baseline [35]. However, our study did not include advanced AD cases, which may explain the lack of observable accumulation in some neocortical regions (e.g., occipital and parietal cortices). Conversely, no measurable change of amyloid-beta load was detected over 1 year, probably due to the limited sample size (*n*=13), short time interval between scans (12 months), and the different accumulation rates of amyloid and tau. Previous studies have shown that the average annual rate of amyloid-beta accumulation measured with PET in the MCI population is around 1-2% [36–39], making it difficult to detect in small samples over 1 year. In contrast, the annual tau accumulation observed in this sub-study ranged from 2 to 6%. Most of the subjects that underwent longitudinal scans had low tau deposition at baseline (Fig. 3, solid dots), suggesting that the expected changes over time in this study population would be small compared to what might be expected with more advanced cases. Indeed, statistically significant changes in this small relatively unaffected cohort indicate the good sensitivity of ^18^F-PI-2620 to detect subtle tau deposition over time.

^18^F-PI-2620 SUVR correlated with some recall-specific domains of the cognitive scores (MMSE (Registration, Recall), ADAS-Cog14 (Delayed word recall, Word recognition), CBB (One-card learning, Identification), and ISLT (Delayed recall)) but also in (MMSE (Registration), ADAS-Cog14 (Word recognition), and CBB (One-card learning, Identification). The correlation pattern was predominantly focused on the mesial temporal cortex, fusiform gyrus, and inferior temporal cortex. Relatively low correlation was found in some neocortical regions such as parietal or frontal cortices and cognitive scores, as reported by Devous et al. (2021) and Ossenkoppele et al. (2016) [3, 40], which can be explained in part by the early population included in this study with very limited neocortical tracer uptake. In the current study, no statistically significant associations were found between cognitive decline and ^18^F-PI-2620 SUVR at baseline due to the limited sample size. As an exploratory analysis and hypothesizing that elenbecestat may not have any effect on cognition, the assessment was expanded by including longitudinal ^18^F-PI-2620 PET of those subjects treated with elenbecestat (*n*=12). In this analysis, decline in MMSE Orientation to place score and ADAS-Cog Delayed word-recall and Total scores 13 and 14 at 1-year follow-up showed the strongest association with tau deposition at baseline in regions with early tau deposition (Supplemental material 4). These results are consistent with previous publications using other tau radioligands where baseline tau load and change in tau load were both significantly associated with changes in cognitive performance [35]. In this regard, as a limitation of the study, it must be noted that given the study’s exploratory nature and the relatively small sample size, *p*-values without correction for multiple comparisons were reported as guidance of the reliability of the results, but they should not be considered as formal hypotheses testing. The findings regarding the association between SUVR and cognitive scores or cognitive decline should be confirmed in studies with larger samples. Finally, SUVR could be biased by the effect of cerebral blood flow changes over time affecting the percent of amyloid and tau load estimates. Several studies reported limited ^18^F-florbetaben SUVR bias when cerebral blood flow changes are small as would be expected in the study population [41, 42]. However, no information is available on the impact of cerebral blood flow on ^18^F-PI-2620 SUVR estimates.

## Conclusion

The findings support the hypothesis that ^18^F-PI-2620 PET imaging of neuropathologic tau deposits may reflect underlying neurodegeneration in AD. Significant correlations were observed with hippocampal volume and CSF biomarkers, and an association between tau and Aβ load as expected from previous publications. Quantifiable increases in ^18^F-PI-2620 SUVR over 1 year were observed in regions with early tau deposition and the results are consistent with the hypothesis that cortical tau is associated with cognitive impairment. This study supports the utility of 18F-PI-2620 PET to assess tau deposits in early AD population. Quantifiable tau load and its corresponding increase in early AD cases could be a relevant target engagement marker for anti-tau but also anti-amyloid clinical trials.

## Supplementary Information

**Additional file 1: Supplemental material 1**. Spaghetti plots showing individual trajectories per participant stratified as low (CL<36) (blue) and elevated (CL≥36) (red) amyloid-beta burden. Gray circles correspond to subjects without longitudinal ^18^F-PI-2620 PET scans.

**Additional file 2: Supplemental material 2**. Scatter plots, linear regression, and Spearman correlation coefficient (ρ) of the cognitive assessment and ^18^F-PI-2620 SUVR at baseline.

**Additional file 3: Supplemental material 3**. Percent tau accumulation (percent ^18^F-PI-2620 SUVR change) over one year follow-up in the subset of subjects in placebo (*n*=15) (top), the subset of subjects with elenbecestat treatment (*n*=12) (center), and the full dataset (*n*=27) (bottom). Statistically significant changes (*p*-value < 0.05) obtained from the linear mixed effect model are marked with an asterisk (*).

**Additional file 4: Supplemental material 4**. Scatter plots, linear regression, and Spearman correlation coefficient (ρ) of the cognitive assessment change and ^18^F-PI-2620 SUVR at baseline of subjects on placebo (red), subjects treated with elenbecestat (blue). Black line and equation correspond to the regression line with the whole dataset.

## Data Availability

The datasets generated and/or analyzed during the current study are not publicly available.
