# Supplementary material for: Evaluation of tau deposition using 18F-PI-2620 PET in MCI and early AD subjects—a MissionAD tau sub-study
Source: Alzheimers Res Ther. 2022 Jul 27;14:105. doi: 10.1186/s13195-022-01048-x (PMC9327167; doi:10.1186/s13195-022-01048-x)
Supplement: Supplementary file 1 — Additional file 1: Supplemental material 1. Spaghetti plots showing individual trajectories per participant stratified as low (CL<36) (blue) and elevated (CL≥36) (red) amyloid-beta burden. Gray circles correspond to subjects without longitudinal 18F-PI-2620 PET scans. [file 13195_2022_1048_MOESM1_ESM.docx]

**Supplemental material 1**. Spaghetti plots showing individual trajectories per participant stratified as low (CL<36) (blue) and elevated (CL≥36) (red) amyloid-beta burden. Gray circles correspond to subjects without longitudinal ^18^F-PI-2620 PET scans.

**
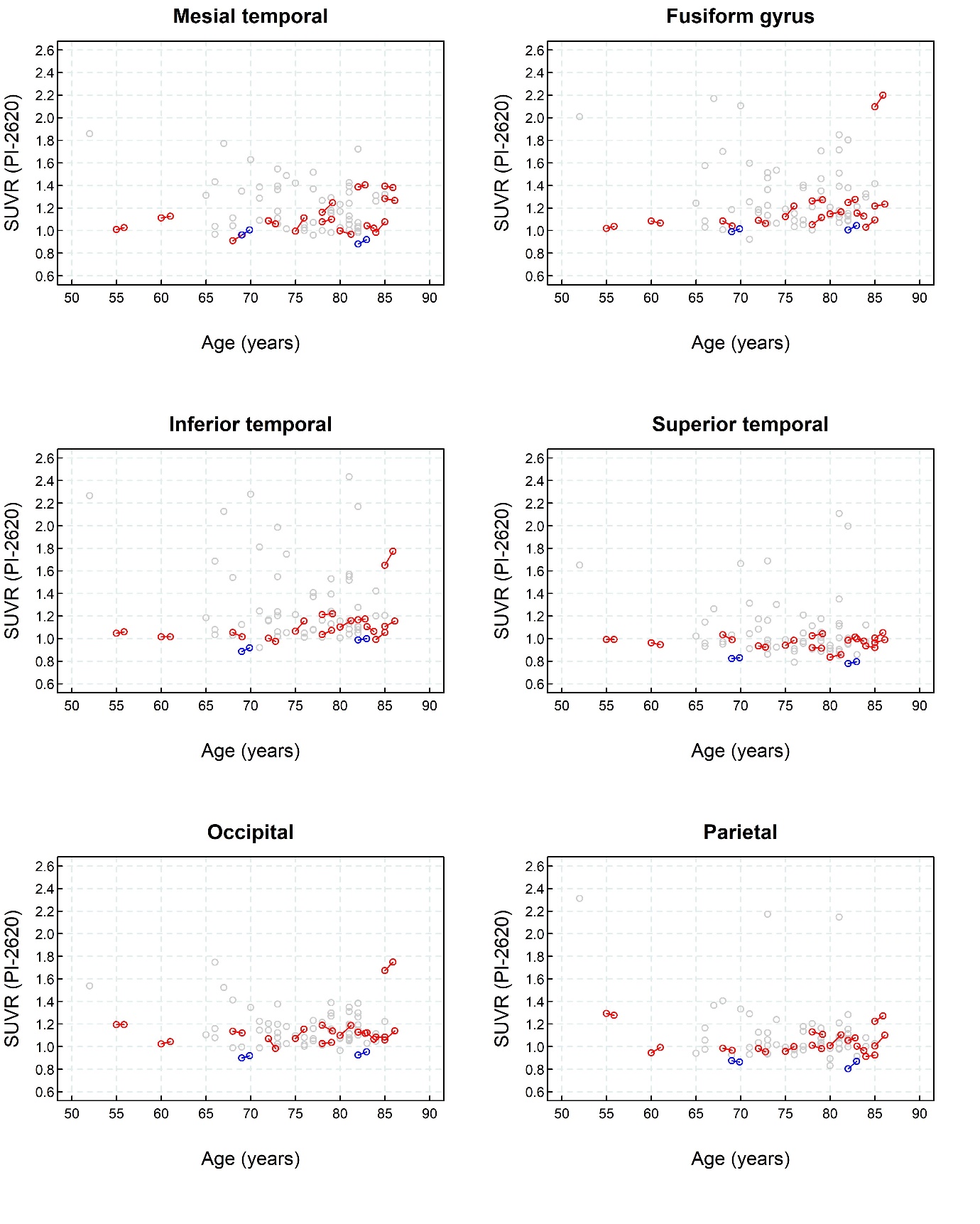
**
