# Supplementary material for: Evaluation of tau deposition using 18F-PI-2620 PET in MCI and early AD subjects—a MissionAD tau sub-study
Source: Alzheimers Res Ther. 2022 Jul 27;14:105. doi: 10.1186/s13195-022-01048-x (PMC9327167; doi:10.1186/s13195-022-01048-x)

**Supplemental material 2**. Scatter plots, linear regression, and Spearman correlation coefficient (ρ) of the cognitive assessment and ^18^F-PI-2620 SUVR at baseline.


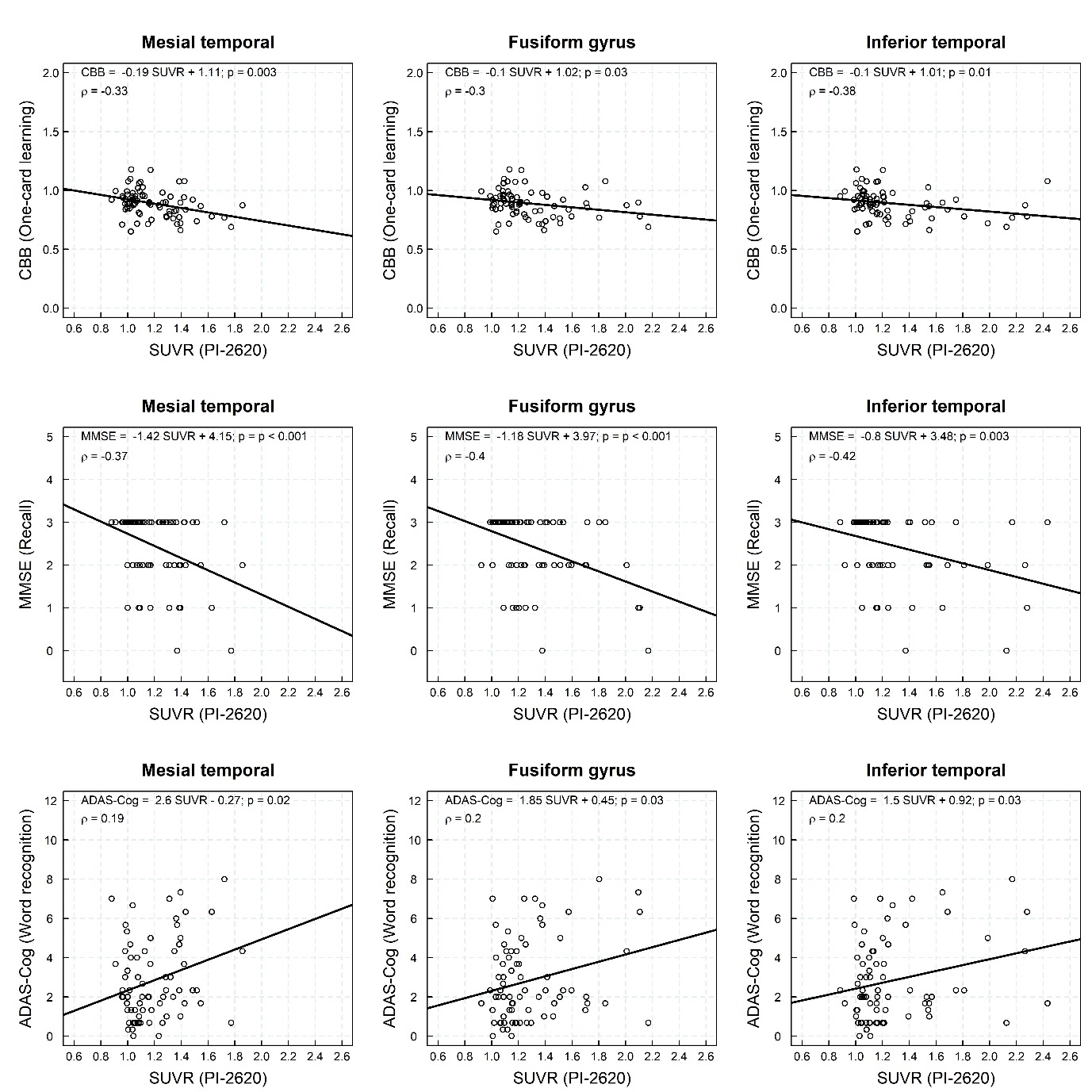

Supplement: Supplementary file 2 — Additional file 2: Supplemental material 2. Scatter plots, linear regression, and Spearman correlation coefficient (ρ) of the cognitive assessment and 18F-PI-2620 SUVR at baseline. [file 13195_2022_1048_MOESM2_ESM.docx]
