# Supplementary material for: Evaluation of tau deposition using 18F-PI-2620 PET in MCI and early AD subjects—a MissionAD tau sub-study
Source: Alzheimers Res Ther. 2022 Jul 27;14:105. doi: 10.1186/s13195-022-01048-x (PMC9327167; doi:10.1186/s13195-022-01048-x)
Supplement: Supplementary file 3 — Additional file 3: Supplemental material 3. Percent tau accumulation (percent 18F-PI-2620 SUVR change) over one year follow-up in the subset of subjects in placebo (n=15) (top), the subset of subjects with elenbecestat treatment (n=12) (center), and the full dataset (n=27) (bottom). Statistically significant changes (p-value < 0.05) obtained from the linear mixed effect model are marked with an asterisk (*). [file 13195_2022_1048_MOESM3_ESM.docx]

**Supplemental material 3**. Percent tau accumulation (percent ^18^F-PI-2620 SUVR change) over one year follow-up in the subset of subjects in placebo (n=15) (top), the subset of subjects with elenbecestat treatment (n=12) (center), and the full dataset (n=27) (bottom). Statistically significant changes (p-value < 0.05) obtained from the linear mixed effect model are marked with an asterisk (*).

**~~
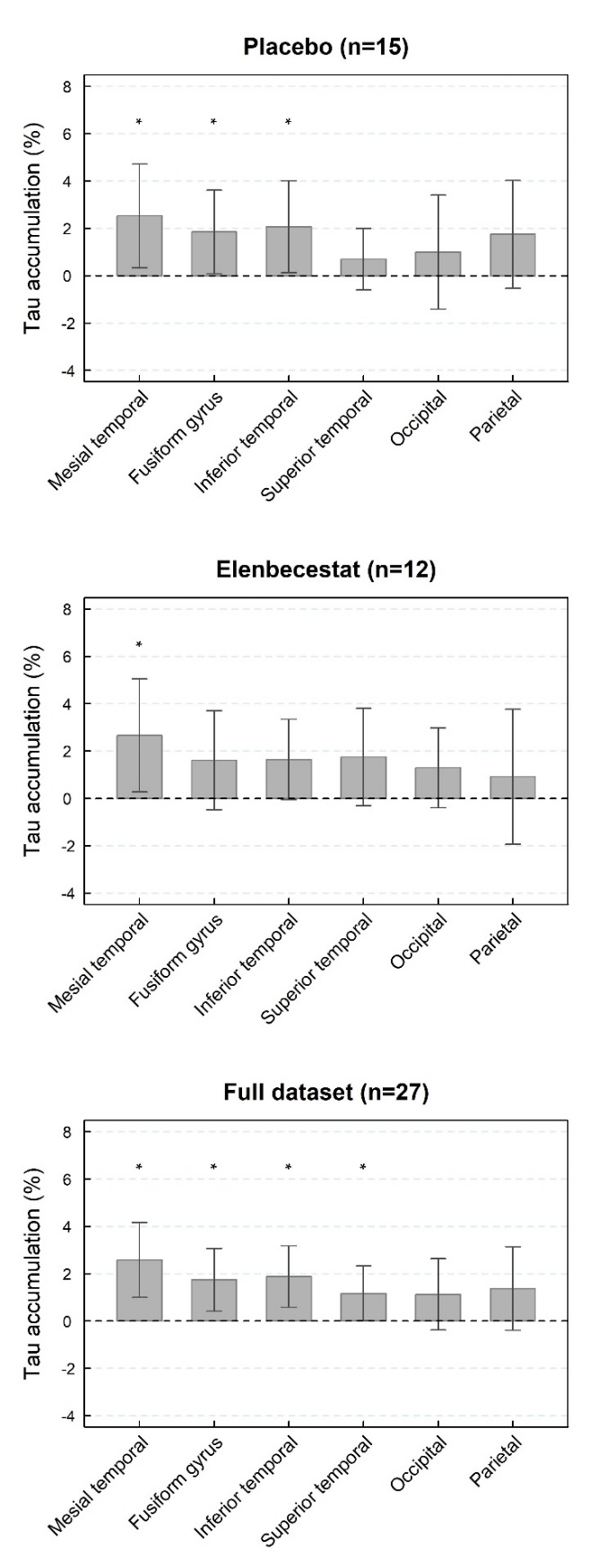
~~**
