# Supplementary material for: Evaluation of tau deposition using 18F-PI-2620 PET in MCI and early AD subjects—a MissionAD tau sub-study
Source: Alzheimers Res Ther. 2022 Jul 27;14:105. doi: 10.1186/s13195-022-01048-x (PMC9327167; doi:10.1186/s13195-022-01048-x)

**Supplemental material 4**. Scatter plots, linear regression, and Spearman correlation coefficient (ρ) of the cognitive assessment change and ^18^F-PI-2620 SUVR at baseline of subjects on placebo (red), subjects treated with elenbecestat (blue). Black line and equation correspond to the regression line with the whole dataset.


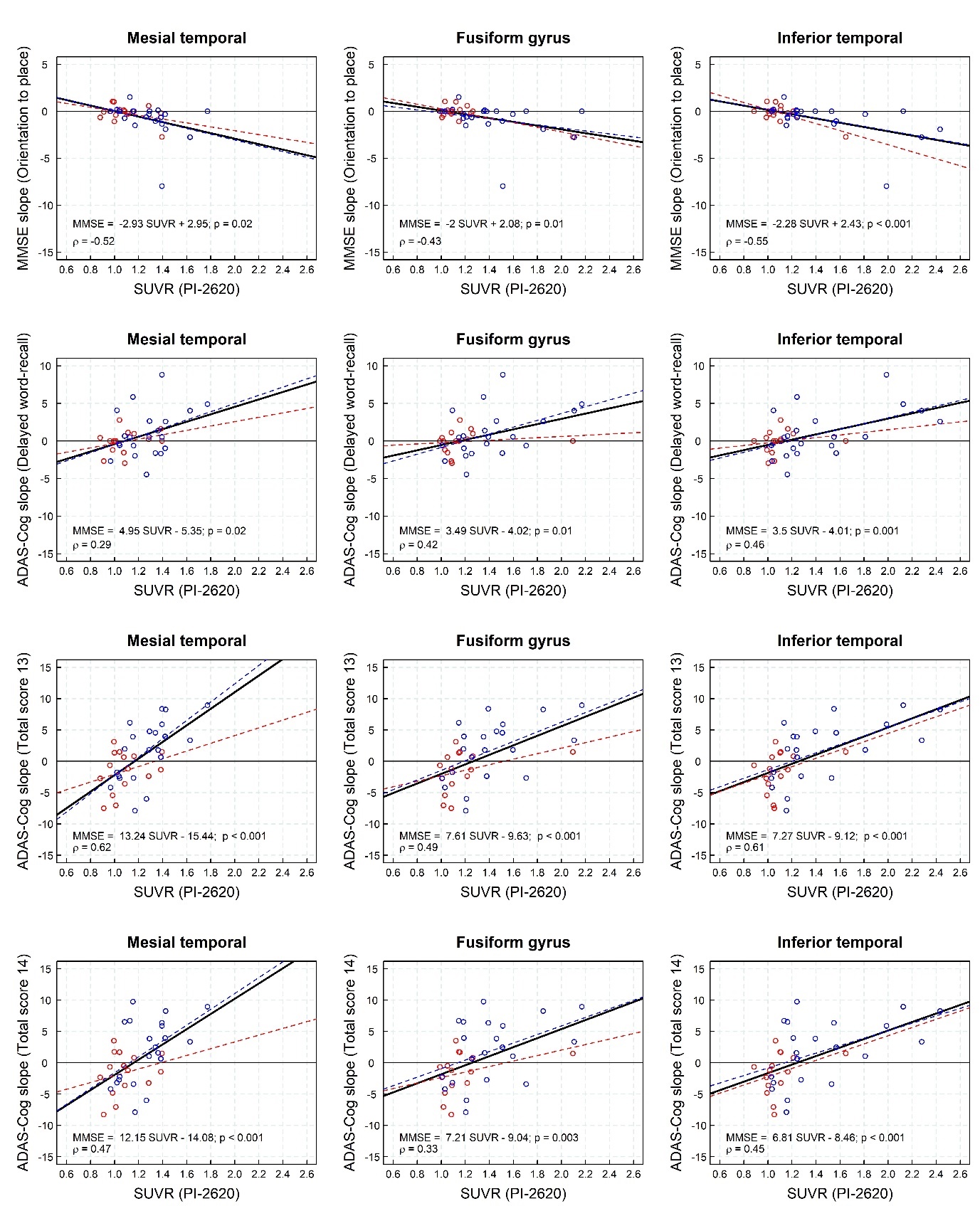

Supplement: Supplementary file 4 — Additional file 4: Supplemental material 4. Scatter plots, linear regression, and Spearman correlation coefficient (ρ) of the cognitive assessment change and 18F-PI-2620 SUVR at baseline of subjects on placebo (red), subjects treated with elenbecestat (blue). Black line and equation correspond to the regression line with the whole dataset. [file 13195_2022_1048_MOESM4_ESM.docx]
